# Optimal Timing of Angiography-Guided Complete Revascularization of Non-Culprit Lesions in STEMI Patients with Multivessel Disease

**DOI:** 10.3390/jcm13175070

**Published:** 2024-08-27

**Authors:** Vincenzo Sucato, Cristina Madaudo, Antonia Marotta, Antonella Ortello, Emmanuele Antonio Camarda, Francesco Comparato, Alfredo Ruggero Galassi

**Affiliations:** Division of Cardiology, Department of Excellence of Sciences for Health Promotion and Mother and Child Care, Internal Medicine and Specialties (ProMISE), University Hospital Paolo Giaccone, University of Palermo, Via Del Vespro n° 129, 90127 Palermo, Italy; cristina.madaudo@unipa.it (C.M.); antonia.marotta@unipa.it (A.M.); antonella.ortello@unipa.it (A.O.); emmanuele.camarda@unipa.it (E.A.C.); francesco.comparato@unipa.it (F.C.);

**Keywords:** STEMI, multivessel disease, coronary artery disease, revascularization, coronary microcirculation

## Abstract

**Background:** There are many questions regarding the optimal approach to treating non-culprit lesions in STEMI patients. Several questions still need to be answered, such as identifying the lesions to be revascularized and the optimal timing. **Methods:** We conducted a single-center analysis. The primary outcome was the incidence of major cardiovascular and cerebral adverse events (MACCE) at 12 months in patients with STEMI and multivessel disease (MVD) who achieved complete revascularization during the index procedure or with a staged procedure. The secondary outcomes were death from any cause, myocardial infarction, target lesion revascularization, stroke, major bleeding events, new angina episodes, new hospitalization, and in-hospital MACCE. **Results:** From January 2021 to December 2022, a total of 230 patients with STEMI underwent primary PCI in our department; 87 patients had MVD. Fifty-nine patients (67.8%) underwent a non-culprit revascularization strategy during the index procedure strategy, and 28 patients (32.2%) during a staged procedure. The incidence of MACCE at 12 months was 11.9% (seven patients) in the index PCI group, compared with 32.1% (nine patients) in the staged PCI group (odds ratio, 3.52; 95% CI, 1.15 to 10.77; *p* = 0.022). In-hospital MACCE occurred in five patients (8.5%) of the index PCI group, compared with seven patients (25%) in the staged PCI group (odds ratio, 3.60; 95% CI, 1.03 to 12.61; *p* = 0.036). A trend towards better outcomes favoring the index PCI group was observed with death from any cause, myocardial infarction, target lesion revascularization, and new angina episodes. **Conclusions:** Better outcomes were evident with an index PCI strategy than with a staged PCI strategy for complete revascularization in patients with STEMI and MVD.

## 1. Introduction

Multivessel disease (MVD) in patients presenting with ST-segment elevation acute myocardial infarction (STEMI) has been a hot interventional research field for years. About 30–40% of patients with STEMI who underwent primary percutaneous coronary intervention (PCI) presented with significant stenosis in one or more non-infarct-related arteries (IRAs) on coronary angiography [1,2].

Despite the need to treat the culprit lesion, revascularization of non-IRA lesions is still controversial in the catheterization laboratory. European guidelines on revascularization published in 2018 recommended that routine revascularization of non-IRA lesions should be considered in patients with MVD before hospital discharge (class of recommendation IIa, level of evidence A), as in all studies then available, PCI of MVD had been performed at that time [3]. However, in the COMPLETE study, PCI of non-IRA lesions in patients randomized to complete revascularization was performed during hospitalization (67% of cases) or after discharge (33% of cases), at a mean time of 23 days from discharge, but within 45 days in all cases, without highlighting any interaction between the effect of treatment and the timing of PCI [4]. European guidelines on acute coronary syndrome (ACS), published in 2023, have overturned this concept. For this reason, in patients with STEMI, complete revascularization is recommended in class I (level of evidence A) during the index procedure or within 45 days. In general, PCI of non-IRA is based on angiographic severity [5].

These recommendations were developed based on several clinical trials that evaluated the revascularization of non-IRA lesions during both the index PCI and staged PCI [4,6,7,8,9].

However, these trials were smaller and often underpowered, and they suffered from several biases. Our study reported a single-center experience addressing different timing strategies for complete non-IRA revascularization.

## 2. Materials and Methods

**Figure 1:**
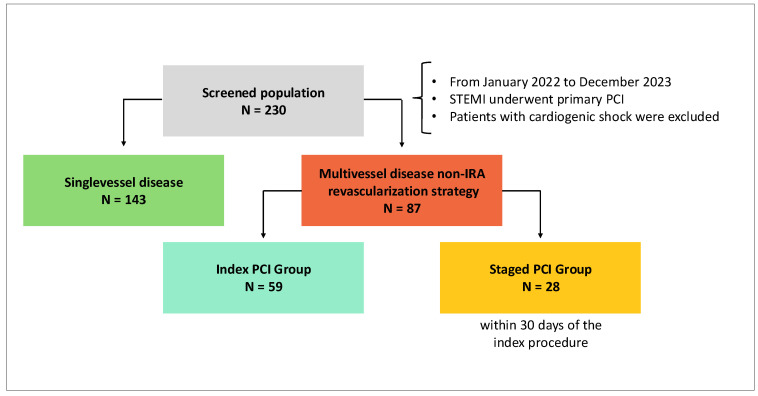
Flowchart of the study group selection process.

We conducted a retrospective analysis including patients diagnosed with STEMI who underwent primary PCI from January 2021 to December 2022 and recovered in the Intensive Care Unit (ICU). Patients with cardiogenic shock were excluded from this analysis. Figure 1 shows the flowchart of the study group selection process. We collected baseline data about the clinical, electrocardiographic, and angiographic presentation; procedural angiographic details; and major adverse events in the hospital. All patients underwent follow-up at 12 months. The primary outcome was the incidence of major cardiac and cerebrovascular events (MACCE, a composite of death from any cause, myocardial infarction, stroke, and major bleeding events) at 12 months. The secondary outcomes were the incidence of every single component of the primary outcome and the incidence of target lesion revascularization (TLR), new angina episodes, and new hospitalization for repeat coronary angiography.

### Statistical Analysis

Continuous variables are expressed as the mean ± standard deviation (SD), median [25th, 75th percentiles], or N (%). Counts and percentages denote categorical variables. All the variables recorded were compared between the two study groups (the index PCI group and the staged PCI group). Student’s *t*-test or the Mann–Whitney test, as appropriate, was used to compare the continuous variables of the two study groups; proportions were compared using the χ^2^-test and Fisher’s exact test.

Univariable Cox regression analysis was performed to identify factors independently associated with MACCE in the study population.

A two-sided *p*-value < 0.05 was considered statistically significant for single tests, whereas, for multiple testing, the significance level was adjusted using the Bonferroni correction. All statistical analyses were performed using SPSS software (Version 29.0, IBM, Armonk, NY, USA).

## 3. Results

From January 2022 to December 2023, 230 patients with STEMI underwent primary PCI in our department. Overall, 69.5% of patients were males, and the age range of the sample was 25 to 92. The total number of STEMI patients with MVD who underwent a non-IRA revascularization strategy was 87 (37.8%).

Of these, 59 patients (67.8%) underwent a non-IRA revascularization strategy during the index procedure strategy (the index PCI group) and 28 patients (32.2%) during a staged procedure within 30 days of the index procedure (the staged PCI group). No differences between the two groups were evident regarding sex, age, or cardiovascular risk factors (Table 1).

**Figure 2:**
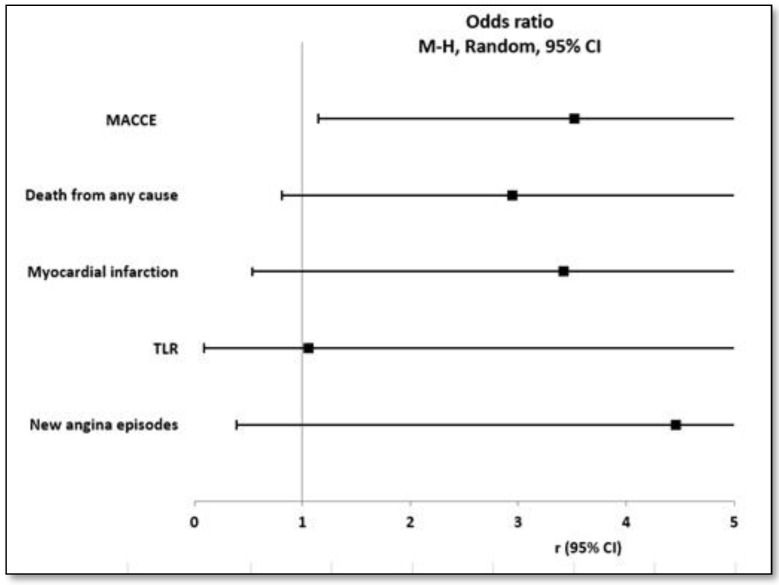
Forest plot of results at 12 months. MACCE: major cardiovascular and cerebral adverse events (i.e., a composite of death from any cause, myocardial infarction, stroke, and major bleeding events); TLR: target lesion revascularization.

Table 2 and Figure 2 show the incidence of outcomes at a median follow-up of 12 months. The incidence of the primary outcome (MACCE, a composite of death from any cause, myocardial infarction, stroke, and major bleeding events) had occurred in nine patients (32.1%) in the staged PCI group, compared with seven patients (11.9%) in the index PCI group (odds ratio, 3.52; 95% CI, 1.15 to 10.77; *p* = 0.022).

**Figure 3:**
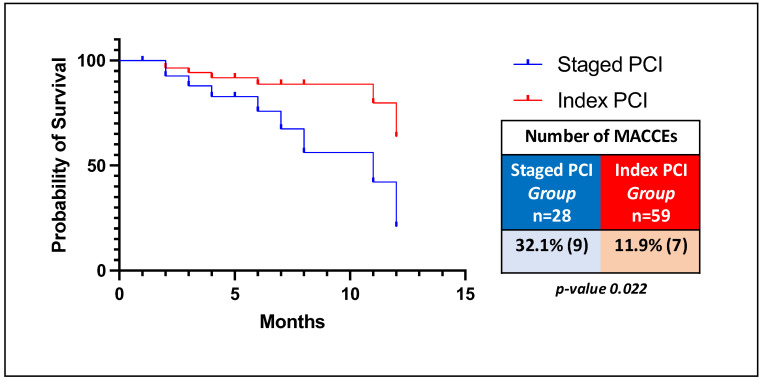
Time-to-event Kaplan–Meier curve: survival free from incidence of MACCEs.

A Kaplan–Meier time-to-event curve (Figure 3) was plotted to show survival free from incidence of MACCEs in the two different groups (index PCI vs. staged PCI).

Regarding the secondary outcomes, death from any cause occurred in five (8.47%) patients in the index PCI group and six (21.4%) patients in the staged PCI group, respectively (odds ratio, 2.95; 95% CI, 0.81 to 10.66; *p* = 0.084). Myocardial infarction occurred in two (3.4%) patients in the index PCI group and in three (10.7%) patients in the staged PCI group, respectively (odds ratio, 3.42; 95% CI, 0.54 to 21.75; *p* = 0.170). Target lesion revascularization occurred in two (3.4%) patients in the Index PCI group and one (3.6%) patient in the staged PCI group, respectively (odds ratio, 1.06; 95% CI, 0.09 to 12.16; *p* = 0.965). New angina episodes occurred in one (1.7%) patient in the index PCI group and two (7.1%) patients in the staged PCI group, respectively (odds ratio, 4.46; 95% CI, 0.39 to 51.43; *p* = 0.193). Two patients (3.4%) in the index PCI group had re-hospitalizations for repeat coronary angiography (*p* = 0.324). No stroke or major bleeding events occurred in either group.

Regarding in-hospital outcomes, MACCE occurred in five patients (8.5%) in the index PCI group and seven patients (25%) in the staged PCI group (odds ratio, 3.60; 95% CI, 1.03–12.61; *p* = 0.036).

## 4. Discussion

The PRAMI trial (Randomized Trial of Preventive Angioplasty in Myocardial Infarction) and CVLPRIT trial (Complete Versus Lesion-Only Primary PCI Trial) are the two most important trials contributing to the evidence in support of an angiography-based approach to complete revascularization [6,7].

The PRAMI study evaluated the benefit of randomly receiving treatment of the culprit lesion (n = 231) or complete revascularization during the index primary PCI in 465 patients with STEMI. The study showed a significant increase in benefit in the group undergoing complete revascularization, enough to stop it prematurely [6].

In the CVLPRIT trial, complete revascularization was based on the angiographic finding of stenosis > 70% in the non-culprit lesion. This trial allowed complete revascularization during the index PCI or hospitalization. The trial enrolled 296 patients with STEMI.

After 1 year of follow-up, the primary endpoint (a composite of all-cause death, recurrent myocardial infarction, heart failure, and ischemia-driven revascularization) was significantly lower in patients randomized to complete compared to culprit-only revascularization (10% vs. 21.2%; HR 0.45, CI: 0.24–0.84; *p* = 0.009) [7].

The COMPLETE trial results have been published to overcome these limits and determine which revascularization strategy should be the standard of care [4]. Along with the composite of cardiovascular death and myocardial infarction, a second co-primary outcome—the composite of cardiovascular death, myocardial infarction, and ischemia-driven revascularization—was assessed in patients with STEMI and MVD who had undergone successful culprit lesion PCI compared to a complete revascularization strategy with PCI of angiographically significant non-culprit lesions or no further revascularization.

The authors demonstrated a statistically significant reduction in the long-term risk (at a median follow-up of 3 years) of cardiovascular death or myocardial infarction in patients with STEMI who underwent a strategy of complete revascularization of the non-IRA lesion in multiple stages compared with a strategy of culprit-lesion-only PCI [4]. The benefit was evident regardless of the intended timing of the non-culprit-lesion PCI (earlier, during the index hospitalization, or later, several weeks after discharge; *p* = 0.62 and *p* = 0.27 for interaction for the first and second co-primary outcomes, respectively). However, even if the evidence is more consistent, several issues still need to be addressed, such as the optimal timing of the revascularization and the correct identification of non-IRA lesions to be treated.

In the COMPLETE trial, no patients underwent complete revascularization during the index PCI. This strategy offers an advantage over a staged procedure regarding days of hospitalization and hospitalization-related adverse outcomes. The results of the COMPLETE study have simplified the choice of the optimal revascularization strategy to address MVD in patients with STEMI. The evidence is strong enough to recommend a complete revascularization of non-IRA lesions in these patients. No differences were found if the revascularization was performed during the index hospitalization or after hospital discharge (within 45 days) [4,10].

However, whether a strategy of complete revascularization during the index PCI could improve outcomes has yet to be well established. In our retrospective analysis, we found a statistically significant reduction in the primary outcome (MACCE, a composite of death from any cause, myocardial infarction, stroke, and major bleeding events at 12 months) in patients who underwent an index procedure strategy compared to the staged PCI strategy. A trend towards better results was also visible in patients who underwent an index procedure approach in terms of death from any cause and myocardial infarction, even if the difference lacked statistical significance.

Our results fit the data in the literature. In the Multistars AMI study, an international, open-label, randomized, non-inferiority trial conducted at 37 centers in Europe, 840 hemodynamically stable patients with STEMI and MVD were randomly assigned to immediate complete revascularization or PCI of the culprit lesion followed within 19–45 days of the index procedure for staged PCI of non-culprit lesions. The primary endpoint (a composite of death from any cause, nonfatal myocardial infarction, stroke, unplanned ischemia-driven revascularization, and hospitalization for heart failure at one year), nonfatal myocardial infarction, and unplanned ischemia-driven revascularization were significantly lower in immediate group (8.5%, 2%, and 4.1%, respectively) compared to the staged group (16.3%, 5.3%, and 9.3%) (hazard ratio, 0.52; 95% confidence interval, from 0.38 to 0.72; *p* < 0.001 for non-inferiority and *p* < 0.001 for superiority) [11]. Although the results are encouraging, the window of 19 to 45 days for staged PCI, along with the exclusion of patients with stent thrombosis, in-stent restenosis, and chronic total occlusion, may also have introduced a bias toward non-inferiority [11].

The BIOVASC study also demonstrated that an immediate complete revascularization strategy was non-inferior to a multi-stage complete revascularization strategy. In addition, this study enrolled patients with ACS, including unstable angina, non-ST-segment elevation myocardial infarction, and STEMI [12].

To date, there is no clarity on the data from meta-analyses on the impact of different revascularization strategies. In a meta-analysis of four trials, Gaffar et al. reported a reduced risk of unplanned repeat revascularization and a trend towards lower short- and long-term risks of major adverse cardiovascular events in patients who underwent a single-stage strategy [13]. However, these results were mainly driven by the results of the SMILE trial and were not associated with a reduction in long-term cardiovascular death [14,15,16,17].

Conversely, in a meta-analysis published by Li et al., better results regarding short- and long-term mortality and a trend towards lower major adverse cardiovascular events were seen in patients who underwent a staged strategy [18].

Some meta-analyses have demonstrated a survival benefit of the complete revascularization approach for STEMI in patients with MVD [19,20]. However, Osman et al. reported no significant differences in endpoints (including CV death) between the two revascularization strategies (RR 0.78; 95% CI, 0.60–1.03; *p* = 0.08); the same was true for MI (RR 0.73; 95% CI, 0.58–1.08; *p* = 0.08) and all-cause death (RR 0.90; 95% CI, 0.73–1.12; *p* = 0.36) in particular [21]. Regarding complications, Ahmad et al. reported a nonsignificant difference in the rate of contrast-induced nephropathy (*p* = 0.152) and bleeding risk (*p* = 0.540) [19].

However, both trials and observational studies were included in this analysis, increasing the risk of possible biases [18].

Choosing a staged strategy may seem useful because the prothrombotic and inflammatory environment in the acute phase of STEMI may lead to an increased incidence of acute stent thrombosis. In addition, the severity of non-culprit lesions may be overestimated during the index PCI due to coronary artery spasm and endothelial dysfunction. [22,23]. Furthermore, in a staged procedure, the surgeon can evaluate the degree of microcirculation dysfunction and coronary vasospasm by evaluating the Index of Microvascular Resistance (IMR) and performing an acetylcholine test [23,24].

However, there are several reasons to prefer a single-stage strategy. Potential benefits of an index procedure strategy may include reduced hospital stays, fewer hospital-related adverse events (such as infections), lower costs, and improved coronary microcirculation. An immediate multivessel PCI approach can also reduce the amount of total contrast volume needed and the duration of radiation exposure. It would avoid the need for an additional arterial puncture and associated complications. Furthermore, the patient would not undergo subsequent revascularization procedures and therefore would not require a second hospital stay, thus potentially reducing the overall length of hospital stay and therefore also the costs for the healthcare system [25].

Another critical question that needs to be addressed when we face the problem is the correct identification of non-IRA lesions that should be treated. In the COMPLETE trial, these lesions were deemed angiographically significant if they were associated with at least 70% stenosis of the vessel diameter on visual estimation or with 50 to 69% stenosis accompanied by a fractional flow reserve (FFR) measurement of 0.80 or less [4].

However, due to the time required, FFR evaluation of non-IRA lesions may not be feasible during the single stage strategy. Moreover, looking at the future, a longer time could be needed due to an increased use of intracoronary imaging techniques. In the management of patients with ACS, the use of intracoronary imaging plays a crucial role by providing detailed information on the atherosclerotic plaque. This resource can be useful in guiding appropriate and personalized treatment by offering insights into the pathology underlying the disease process. Intravascular ultrasound (IVUS) and optical coherence tomography (OCT) are complementary techniques that allow better identification of dimensional and composition characteristics of coronary lesions, leading to better stent sizing and apposition [22]. OCT has a higher resolution, making it more suitable than IVUS for detailed visualization and identification of vulnerable plaque features. However, IVUS-NIRS does not require image interpretation to detect lipid core plaques and allows automated quantification, making it useful without in-depth expertise. Despite the superior detail of OCT, its limited tissue penetration and the need for contrast injection may limit its use, particularly in patients with poor renal function, in whom IVUS would be preferable [22].

However, PCI of the IRA should not be deferred based on invasive epicardial functional assessment in patients with ACS (class of recommendation III, level of evidence C). The coronary microcirculation begins to recover within 24 h of PPCI, and acute functional assessment of the IRA may underestimate the true hemodynamic severity of the coronary stenosis [26]. A simple risk score developed by Schamroth et al. based on three angiographic characteristics could help clinicians choose the non-IRA lesions to be revascularized in case the FFR or revascularization is unavailable during the index procedure [27]. However, this score needs more extensive and external validations in order to enter common practice.

Furthermore, recent studies have shown the crucial role of inflammation in cardiac events [28]. The underlying mechanisms include systemic inflammatory responses and endothelial dysfunction. Myocardial infarction is associated with a systemic and local inflammatory response with edema, and higher non-infarcted myocardium T2 values on cardiac magnetic resonance (CMR) after STEMI are independently associated with worse cardiovascular outcomes [29]. This phenomenon suggests that persistent inflammation may contribute to cardiac vulnerability, regardless of the site of the primary infarction [30].

### Limitations of the Study and Future Directions

The main limitations of our study are the retrospective design and the small sample size, which made our results only hypothesis-generating. Larger studies and randomized controlled trials are needed to deepen our understanding of this phenomenon and explore its practical implications in different settings and to understand which of the two strategies might work better. There are some ongoing trials that may provide more precise answers on the efficacy and safety of an immediate complete revascularization strategy versus a staged PCI strategy for non-IRA patients (NCT04968808) and others comparing clinical outcomes between IVUS-guided treatment decisions and FFR-guided treatment decisions for non-IRA lesions in patients with STEMI and MVD (NCT05812963).

## 5. Conclusions

Among hemodynamically stable patients with STEMI and MVD, a trend toward better outcomes was observed in favor of the immediate multivessel PCI group. The incidence rates of both in-hospital MACCE and MACCE at 12 months were lower in the index PCI group than in the staged PCI group (*p* = 0.036 and *p* = 0.022, respectively). The incidence rates of all-cause death, myocardial infarction, target lesion revascularization, and new episodes of angina were lower in STEMI patients with MVD undergoing a non-IRA revascularization strategy during the index procedure. A deeper understanding of these processes could lead to targeted therapeutic strategies to reduce the risk of future cardiac events.

## Figures and Tables

**Table 1:** Baseline characteristics.

Variables	Screened Population n. (%)	Enrolled Population n. (%)	Index PCI n. (%)	Staged PCI n. (%)	*p* Value
	n = 230	n = 87	n = 59	n = 28	
Male sex	160 (69.5)	61 (70.1)	41 (69.5)	20 (71.4)	0.85
Age	58.5 ± 10	61 ± 14	60 ± 14	62 ± 13	0.52
Hypertension	146 (63.4)	64 (73.5)	43 (72.8)	21 (75)	0.83
Diabetes	64 (27.8)	27 (31)	17 (28.8)	10 (35.7)	0.58
Smoking	123 (53.4)	53 (61)	37 (62.7)	16 (57)	0.61
Dyslipidemia	75 (32.6)	32 (36.8)	21 (35.9)	11 (39.2)	0.73
History of CV diseases	103 (47.7)	40 (46)	29 (49.1)	11 (39.2)	0.38
Multivessel disease	97 (42.1)	87 (100)	59 (100)	28 (100)	NS
Non-IRA revascularization strategy	87 (37.8)	87 (100)	59 (100)	28 (100)	NS

CV: CardioVascular; IRA: infarct-related artery; PCI: percutaneous coronary intervention.

**Table 2:** Index PCI vs. staged PCI: median follow-up at 12 months.

	Enrolled Population n. (%)	Index PCI n. (%)	Staged PCI n. (%)	OR	95% CI	*p* Value
	n = 87	n = 59	n = 28			
MACCE	16 (18.4)	7 (11.9)	9 (32.1)	3.52	1.25 to 10.77	0.022
Death from any cause	11 (12.6)	5 (8.47)	6 (21.4)	2.95	0.81 to 10.66	0.084
Myocardial infarction	5 (5.7)	2 (3.4)	3 (10.7)	3.42	0.54 to 21.75	0.170
TLR	3 (3.5)	2 (3.4)	1 (3.6)	1.06	0.09 to 12.16	0.965
New angina episodes	3 (3.5)	1 (1.7)	2 (7.1)	4.46	0.39 to 51.43	0.193
New hospitalization	2 (2.3)	2 (3.4)	0	-	-	0.324

CI: confidence interval; MACCE: major cardiovascular and cerebral adverse events (i.e., a composite of death from any cause, myocardial infarction, stroke, and major bleeding events); OR: odds ratio; PCI: percutaneous coronary intervention; TLR: target lesion revascularization.

## Data Availability

The data presented in this study are available on request from the corresponding author due to privacy.
